# Microarray dataset of Jurkat cells following miR-93 over-expression

**DOI:** 10.1016/j.dib.2016.06.001

**Published:** 2016-06-20

**Authors:** Silvia Gioiosa, Lorena Verduci, Gianluca Azzalin, Claudia Carissimi, Valerio Fulci, Giuseppe Macino

**Affiliations:** Dipartimento di Biotecnologie Cellulari ed Ematologia, “Sapienza” Università di Roma, Italy

**Keywords:** miR-93, T-lymphocytes, Homo sapiens, Microarray

## Abstract

The dataset presented here represents a microarray experiment of Jurkat cell line over-expressing miR-93 after lentiviral transgenic construct transduction. Three biological replicates have been performed.

We further provide normalized and processed data, log2 Fold Change based ranked list and GOterms resulting table. The raw microarray data are available in the ArrayExpress database (www.ebi.ac.uk/arrayexpress) under accession number ArrayExpress: E-MTAB-4588.

**Specifications Table**

**Table:** 

Subject area	*Biology*
More specific subject area	*Molecular biology*
Type of data	*Text, excel file, table*
How data was acquired	*Affymetrix Human Genome HG U133 Plus 2.0 array*
Data format	Raw, processed
Experimental factors	*miR-93 was over-expressed in Jurkat cells by transduction with a lentiviral transgenic construct encoding for miR-93 under a PGK promoter. A cognate vector encoding for a control hairpin was used to generate control cell line.*
Experimental features	*We analyzed the whole-transcript expression profiling of Jurkat cells (T-ALL cell line) over-expressing miR-93 using a microarray experiment.*
Data source location	*University of Rome, “La Sapienza”, Rome, Italy*
Data accessibility	Data is within this article and microarray data are available through Array Express repository (Accession Number ArrayExpress: E-MTAB-4588)

**Value of the data**

• A global gene expression analysis of human Jurkat cells (T-ALL cell line) over-expressing miR-93.
• miR-93 is frequently dysregulated in several human malignancies. This dataset can represent a further insight into gene expression changes and GO Terms associated with miR-93 over-expression.
• These data can represent a benchmark for further experiments in order to clarify the meaning of the biological processes identified by the in silico data analysis.

## 1 Data

The Affymetrix mRNA profile data are provided as.CEL files deposited on Array Express (http://www.ebi.ac.uk/arrayexpress/arrays/E-MTAB-4588). RNA was extracted from miR-93 over-expressing Jurkat cells (pRRL-93) and matched control cell line (pRRL-Ctrl). Three biological replicates have been performed. Data have been normalized and processed as described in the following section. The attached excel file (miR93_all_filtered_genes.xls) represents the filtered datasest with relative adjusted *p*-values.

## 2 Experimental design, materials and methods

### 2.1 Cell and culture conditions

Jurkat cells were purchased from Deutsche Sammlung von Mikroorganismen und Zellkulturen (DSMZ) and cultured in RPMI-1640 Medium (SIGMA) supplemented with 10% fetal bovine serum (SIGMA), at 37 °C in a 5% CO_2_ atmosphere.

### 2.2 Construction of a lentiviral vector expressing miR-93

PGK-miR93 expression construct was obtained amplifying a region of about 200 nt containing miR-93 precursor, from human genomic DNA. We used the following primers designed to incorporate RsrII and XbaI restriction sites and 6 bp of extra random sequence to aid in restriction digestion: 5′CGCGCGCGGACCGCCCACTTCTTAACCTTC-3′ (forward), 5′GCGCGCTCTAGAGAGTTCAGCTGTCCTGTG-3′ (reverse). The amplified region was cloned into the RsrII NheI sites of #1074.1071.hPGK.GFP.WPRE.mhCMV.dNGFR.SV40PA (kindly provided by Prof Luigi Naldini). PGK-control was obtained by substituting miR-93 precursor fragment (RsrI–NheI) with a sequence encoding for an hairpin yielding a 22-mer RNA designed to lack homology to any human gene. The lentiviral vector expressing miR-93 and the packing vectors pLP1, pLP2, and pLP/VSVG were co-transfected into HEK293T cells, and culture medium was collected at 48 h. The medium was filtered through 0.45-μm pore nitrocellulose filters and then centrifuged in a Beckman ultracentrifuge (Beckman, OptimaTM LE-80K) at 25,000 rpm for 2 h at 4 °C. The precipitate was re-suspended in RPMI complete medium. The viral supernatant and Polybrene 8 μg/ml were added to the Jurkat cells.

### 2.3 mRNA microarray data normalization and processing

Three biological replicates for each sample were hybridized on the array *Human 2.0 ST microarrays*, Affymetrix, Santa Clara, CA. Affymetrix.

CEL files were separately processed for each replicate and experimental condition using the Robust Multi-array Average (RMA) procedure with quantile normalization, log2 transformation and background subtraction, as implemented in the *rma* function from R/BioConductor [1] package *oligo*.

Before calling the differentially expressed genes (DEGs), gene probes were filtered to remove (a) nonspecific probes and probes lacking a reference ID in public information repositories; (b) probes showing minimal variation across samples; and (c) probes with constant low expression across samples.

Particularly, as supplied in the R/BioConductor [1] package *genefilter*, the function *nsFilter* was applied to the normalized expression values with default arguments in order to:

(1) remove Affymetrix control probe sets and features without an Entrez Gene ID annotation; and
(2) probe sets were required to have interquartile range (IQR) of their log2-transformed values at least equal to 0.5;

Moreover, the function *kOverA* from the package *genefilter* was applied in order to remove probe sets marked by low or null expression in total RNA samples (i.e.: require expression value >log 2(100) in both miR-93 over-expressing samples and control samples).

Therefore, we ended up with a list of 6559 filtered probe sets. The *limma* package from R/BioConductor [1] open source software for bioinformatics was used to calculate moderated *t*-statistics (based on the empirical Bayes approach) to identify DEGs between the miR-93 over-expressing group and control group.

Because of multiple hypothesis testing, *p*-values were adjusted by the false discovery rate (FDR) method (miR93_all_filtered_genes.xls).

### 2.4 GO terms analysis

Basing on the log2 Fold Change values, a ranked list of negatively regulated genes was produced. This list has been submitted into the GOrilla software tool [2] as single ranked list of genes (miR93.negative.ranked.list.txt) setting a *p*-value cutoff of 10E-6 (Table1_GOTERMS_Negative_ranked_list.png).
